# Phenytoin as seizure prophylaxis in hematopoietic stem cell transplantation with busulfan conditioning

**DOI:** 10.3389/fneur.2022.928550

**Published:** 2022-08-22

**Authors:** R. S. Germeraad, A. M. P. Demandt, R. P. W. Rouhl

**Affiliations:** ^1^Department of Neurology, Maastricht University Medical Center, Maastricht, Netherlands; ^2^Division of Hematology, Department of Internal Medicine, Maastricht University Medical Center, Maastricht, Netherlands; ^3^School for Mental Health and Neuroscience, Maastricht University, Maastricht, Netherlands; ^4^Academic Center for Epileptology Kempenhaeghe/Maastricht University Medical Centre, Maastricht, Netherlands

**Keywords:** busulfan, phenytoin (PHT), seizure, acute symptomatic seizure, seizure prophylaxis

## Abstract

**Background:**

Phenytoin is widely used as primary seizure prophylaxis in hematopoietic stem cell transplantation in patients undergoing myeloablative conditioning with busulfan. Because of the negative side effects of phenytoin, we abandoned phenytoin use in these patients. To assess the effect of this change, we performed a retrospective cohort study on all patients receiving busulfan.

**Methods:**

We included 139 patients who underwent conditioning with busulfan for hematopoietic stem cell therapy. We registered the use of phenytoin, as well as the occurrence of seizures, until 7 days after busulfan administration. We compared seizure incidence between patients who received phenytoin and those who did not.

**Results:**

Of the 43 patients who received phenytoin prophylaxis, four patients (9.3%) had a seizure during the conditioning regimen, of which two patients had cerebral non-Hodgkin lymphoma. Furthermore, all these 4 patients had very high levels of phenytoin (intoxication). Of the 96 patients that did not receive phenytoin prophylaxis, three patients (3.1%) had a seizure, and one of these patients had an undefined cerebral lesion. Phenytoin did not relate to seizure prevention in a logistic regression analysis.

**Conclusion:**

We conclude that phenytoin prophylaxis in patients treated with busulfan is obsolete and possibly harmful, as phenytoin intoxication can occur. We recommend discontinuing the use of phenytoin as primary seizure prophylaxis in these patients.

## Introduction

Busulfan is an alkylating agent, introduced in 1980 by Santos et al. as a myeloablative regimen in combination with cyclophosphamide in hematopoietic stem cell transplantation (1). Busulfan is a small lipophilic molecule, which easily crosses the blood-brain barrier (2). Treatment with busulfan may lead to organ toxicity in about 10% of the patients (3). Moreover, neurotoxicity can occur with the use of busulfan, and it is likely caused by high levels of busulfan in the central nervous system due to its ability to cross the blood-brain barrier (4). Seizures are considered a common (in ~10% of patients), and possibly preventable, side-effect of busulfan treatment (5, 6).

Phenytoin has been widely used for many years as a drug to prevent seizures induced by busulfan, although a double-blind randomized clinical trial to prove effectivity is missing (4, 7). Despite its effectiveness as an antiepileptic drug, phenytoin has its drawbacks, among others the non-linear pharmacokinetic metabolism, making the elimination very unpredictable with possible consequent toxicity, as well as bothersome side effects (nystagmus, tremors, and myoclonus) (8, 9). Furthermore, phenytoin can cause serious drug-drug interactions, a.o. with busulfan and cyclophosphamide; however, the clinical significance of this interaction is probably negligible (10, 11). Given these factual and possible drawbacks, the benefit-risk ratio of adding phenytoin to busulfan is not clear, and, as we considered that the risks outweighed the benefit (preventing seizures), we decided to stop giving phenytoin to our patients treated with busulfan.

In the current study, we aim to investigate the effect of the discontinuation of phenytoin as primary seizure prophylaxis in busulfan treatment. We performed a retrospective cohort study at our center on a population of patients who underwent hematopoietic stem cell transplantation with a busulfan-based myeloablative stem conditioning regimen.

## Methods

We included 139 adult patients who underwent a myeloablative stem cell transplantation with busulfan-based conditioning between March 2008 and January 2019 at Maastricht University Medical Center (MUMC+). All patients received the busulfan conditioning regime for three consecutive days. Depending on the hematological diagnosis, other chemotherapeutic drugs were also used in the regimen (e.g., fludrarabine and anti-thymocyte globulin for myelofibrosis; fludrarabine for chronic myelocytic leukemia, cyclophosphamide in acute lymphocytic, and myelocytic leukemia). In the period that patients received busulfan, patients either received phenytoin to prevent epileptic seizures or (after we decide to stop using phenytoin in January 2013) no prophylaxis with anti-epileptic drugs at all.

Using the electronic patient files, we assessed the occurrence of seizures. This was done by RG, and all descriptions of episodes with possible epileptic origin were double-checked by RR for classification as an actual seizure. We also examined possible causative factors of seizures, like known brain tumors or other cerebral lesions, and electrolyte and/or metabolic disturbances. This assessment was done in consensus by two clinical experts, one in epilepsy (RR) and one in hematology and hematopoietic stem cell transplantation (AD).

The medical ethical committee approved this retrospective study, which has no obligations to the Dutch law for medical research on patients.

### Statistical analysis

We compared parameters (mainly proportions) in patients who received phenytoin prophylaxis to those without using Chi-Square and Fisher's exact test (whichever was appropriate). Additionally, we performed a logistic regression analysis to evaluate the influence of several factors, such as phenytoin prophylaxis, busulfan dosage, sex, and age on the occurrence of seizure events. We considered *p*-values of < 0.05 to be statistically significant. The statistical analysis was performed with SPSS Statistics version 25.

## Results

Patient characteristics can be found in Table 1. For the busulfan conditioning regimen, 1 mg/kg (orally) or 0.8 mg/kg (intravenously) four times a day was used.

**Table 1:** Patient characteristics.

	**Phenytoin**	**No phenytoin**
	**(*N* = 43)**	**(*N* = 96)**
Male sex	21 (49%)	54 (56%)
Age (years) mean ± SD	47 ± 15.9	50 ± 13.3
**Diagnosis**		
AML^a^	29	57
CML^b^	1	2
Myelofibrosis	1	20
ALL^c^	6	8
Other	6	9
**Stemcell source**		
Autologous	10	51
Allogeneic	33	45
**Busulfan conditioning regime**		
0.8 mg/kg (IV)	0	89
1 mg/kg (oral)	43	7
Mortality	20 (46%)	33 (34%)

a *AML, Acute myeloid leukemia*.

b *CML, Chronic myeloid leukemia*.

c *ALL, Acute lymphoid leukemia*.

In patients receiving phenytoin, the dose was 1.25 mg/kg and given every 6 h, starting 1 or 2 days before, or on the same day of the start of the busulfan regime, until the end of the busulfan regime. All patients took their phenytoin orally. Of the 43 cases that were given with prophylactic phenytoin, four patients (9.5%) had a seizure, of whom two had a cerebral non-Hodgkin lymphoma as a likely contributing cause to the seizure. The seizures occurred between days 3 and 9 after the start of the busulfan regime. Of the 96 patients that did not receive phenytoin, 3 patients (3.1%) had a seizure of whom one patient had cerebral lesions of unknown origin as contributing cause to the seizure. The seizures happened between 3 and 5 days after the start of the busulfan regime. No significant difference in the incidence of seizures was found between both groups (*p* = 0.203, Fisher's exact test). When considering only patients with seizures with an unknown cause (seizures not related to an intracranial lesion), we could not demonstrate significant differences in seizure occurrence between patients with and without prophylaxis (see Table 2).

**Table 2:** Incidence of epileptic seizures with unknown cause in both groups.

	**Epileptic**	**No epileptic**	**Total**
	**seizure**	**seizure**	
Phenytoin prophylaxis	2 (4.7%)^*^	41 (95.3%)	43
No phenytoin prophylaxis	2 (2.1%)^*^	94 (97.9%)	96
Total	4	135	139

* *p = 0.36*.

We also assessed possible confounders in a logistic regression analysis with seizure occurrence as the dependent variable. The dosage of busulfan (which was different when given IV or orally), phenytoin prophylaxis or not, age, and sex did not relate to the occurrence of seizures.

Regarding the safety of phenytoin use, we found that all four patients treated with phenytoin with a seizure had phenytoin intoxication (levels were 21 μg/ml or higher). All other patients in the phenytoin group of whom the phenytoin levels were recorded had therapeutic (or higher) levels (average 15.9 μg/mL or higher), and still, four more patients had clinical signs of a phenytoin intoxication (confirmed by the levels) but without seizures.

## Discussion

In our retrospective study, we could not demonstrate any positive effect of phenytoin on the prevention of seizure occurrence in patients treated with busulfan. In contrast, these patients were exposed to risks, like phenytoin intoxication.

Phenytoin's therapeutic range is between 10 and 20 μg/ml, and elevated levels may lead to severe side effects, such as cerebellar syndrome, while coma may occur with serum levels higher than 40 μg/ml (8, 12). In previous studies, the prevalence of seizures (without seizure prophylaxis) was 1–10% of patients treated with busulfan (4, 13). Our numbers were similar, as we found that 3.1% of patients with busulfan conditioning without phenytoin had an epileptic seizure. Thus, seizure risks do not seem to change over time. However, we found that the seizure risk in the phenytoin-treated group was similar to the non-treated group, leading to the conclusion that phenytoin has no benefit in preventing seizures in these patients. Our findings, therefore, support the decision to stop adding phenytoin to busulfan regimes.

Other anti-epileptic drugs than phenytoin could have presented a more positive picture. Phenytoin (in contrast to other, and newer, anti-epileptic drugs) has an unfavorable side effect profile and narrow therapeutic window, which can easily lead to high levels and intoxication, which, in turn, might cause seizures as well (8). Other anti-epileptic drugs have been assessed for the primary prevention of seizures in busulfan treatments. E.g., Levetiracetam might be a better candidate for primary seizure prevention, though the published studies are not placebo-controlled or randomized. The benefit-risk ratio of newer agents like levetiracetam may be better in these vulnerable patients (7, 14, 15) E.g., levetiracetam has far fewer drug-drug interactions, while showing lesser side effects (15). However, in our study, we show that only two out of the 96 patients who received no prophylaxis, experienced seizures. Providing a prophylactic drug to patients could be beneficial, but even if the effectivity of the anti-epileptic would be 100%, the number needed to be treated is high (in our population 96, to prevent two patients having a seizure). Given these low frequencies, a double-blind randomized trial will not be feasible. Therefore, it remains at the discretion of the treating medical team whether primary seizure prophylaxis will be given (preferably with a newer anti-epileptic drug like levetiracetam) or not.

Despite our important findings, our study has some limitations. First, this was a retrospective study performed in a single center, leading to possible bias in reporting and underreporting seizures and seizure-like events. Also, absolute seizure event numbers were low, both in prophylactically treated patients, as well as in non-treated patients. Of course, this is not beneficial for the power of this study, however, there is no large clinical benefit for the patients treated with phenytoin (we even saw some adverse effects). Secondly, the phenytoin levels were not measured in all the patients leading to missing data in patients without seizures and doing well on phenytoin, nor were busulfan levels measured to assess the effect of possible pharmacokinetic interaction, or to assess the effects of the change of formulation over time from intravenous to oral administration. However, as we mainly focussed on phenytoin treatment and seizure prophylaxis, this was less relevant.

## Conclusion

We found no benefit of phenytoin use in patients treated with busulfan, and we recommend a reconsideration of the standard practice of giving phenytoin as primary prophylaxis in busulfan-based conditioning regimens in hematopoietic stem cell transplantation.

## Data availability statement

The raw data supporting the conclusions of this article will be made available by the authors, without undue reservation.

## Ethics statement

The studies involving human participants were reviewed and approved by METC MUMC+. Written informed consent for participation was not required for this study in accordance with the national legislation and the institutional requirements.

## Author contributions

RG collected the data, performed statistical analysis, and drafted the manuscript. AD supervised the study and revised the manuscript for intellectual and scientific content. RR designed and supervised the study and revised the manuscript for intellectual and scientific content. All authors contributed to the article and approved the submitted version.

## Conflict of interest

The authors declare that the research was conducted in the absence of any commercial or financial relationships that could be construed as a potential conflict of interest.

## Publisher's note

All claims expressed in this article are solely those of the authors and do not necessarily represent those of their affiliated organizations, or those of the publisher, the editors and the reviewers. Any product that may be evaluated in this article, or claim that may be made by its manufacturer, is not guaranteed or endorsed by the publisher.
